# TLR1/TLR2 Heterodimers Play an Important Role in the Recognition of *Borrelia* Spirochetes

**DOI:** 10.1371/journal.pone.0025998

**Published:** 2011-10-05

**Authors:** Marije Oosting, Hadewych ter Hofstede, Patrick Sturm, Gosse J. Adema, Bart-Jan Kullberg, Jos W. M. van der Meer, Mihai G. Netea, Leo A. B. Joosten

**Affiliations:** 1 Department of Medicine, Radboud University Nijmegen Medical Centre, Nijmegen, The Netherlands; 2 Nijmegen Institute of Infection, Inflammation and Immunity (N4i), Radboud University Nijmegen Medical Centre, Nijmegen, The Netherlands; 3 Department of Microbiology, Radboud University Nijmegen Medical Centre, Nijmegen, The Netherlands; 4 Department of Tumor Immunology, Radboud University Nijmegen Medical Centre, Nijmegen, The Netherlands; Universite de la Mediterranee, France

## Abstract

After infection with *Borrelia* species, the risk for developing Lyme disease varies significantly between individuals. Recognition of *Borrelia* by the immune system is mediated by pattern recognition receptors (PRRs), such as TLRs. While TLR2 is the main recognition receptor for *Borrelia* spp., little is known about the role of TLR1 and TLR6, which both can form functionally active heterodimers with TLR2. Here we investigated the recognition of *Borrelia* by both murine and human TLR1 and TLR6. Peritoneal macrophages from TLR1- and TLR6- gene deficient mice were isolated and exposed to *Borrelia*. Human PBMCs were stimulated with *Borrelia* with or without specific TLR1 and TLR6 blocking using specific antibodies. Finally, the functional consequences of TLR polymorphisms on *Borrelia*-induced cytokine production were assessed. Splenocytes isolated from both TLR1−/− and TLR6−/− mice displayed a distorted Th1/Th2 cytokine balance after stimulation with *B.burgdorferi*, while no differences in pro-inflammatory cytokine production were observed. In contrast, blockade of TLR1 with specific neutralizing antibodies led to decreased cytokine production by human PBMCs after exposure to *B.burgdorferi.* Blockade of human TLR6 did not lead to suppression of cytokine production. When PBMCs from healthy individuals bearing polymorphisms in TLR1 were exposed to *B.burgdorferi*, a remarkably decreased *in vitro* cytokine production was observed in comparison to wild-type controls. TLR6 polymorphisms lead to a minor modified cytokine production. This study indicates a dominant role for TLR1/TLR2 heterodimers in the induction of the early inflammatory response by *Borrelia* spirochetes in humans.

## Introduction

Ticks of the *Ixodes* family are able to transmit bacteria of the *Borrelia burgdorferi* sensu lato family, which causes Lyme Disease [1]. Within this family, three species are described to be pathogenic, namely *B.burgdorferi* sensu stricto, *B.afzelii,* and *B.garinii*, which are differentially distributed between the United States and Europe. Clinical signs that develop after infection with *Borrelia* spirochetes are diverse, ranging from skin abnormalities (erythema migrans) to arthritis or carditis [2]. Infection with *Borrelia* results in release of inflammatory mediators and recruitment of inflammatory cells to the site of infection [3], [4]. To induce inflammation, recognition of the bacteria by pattern recognition receptors (PRRs) is necessary.

Distinct classes of PRRs have been described, including C-type lectins (CLRs), NOD-like receptors (NLRs), and Toll-like receptors (TLRs). TLR4, the main receptor for bacterial lipopolysaccharides [5], has been shown not to be involved in the recognition of *Borrelia* [6]. On the other hand, TLR2 plays an important role in the recognition of *Borrelia* components. It has been demonstrated that TLR2-knockout mice produced significantly lower concentrations of antibodies against *Borrelia* after immunization with OspA [7]. TLR2 knockout mice harbor higher amounts of *Borrelia* spirochetes at the site of inflammation than wild-type animals and hence exhibit more cell influx in infected joints [8], [9]. In humans, TLR2 can recognize several components of *Borrelia* [10], [11], and TLR2 blockade in human peripheral blood mononuclear cells (PBMCs) resulted in decreased cytokine production after exposure to intact *Borrelia* spirochetes [6]. In order to recognize its ligands, TLR2 forms heterodimers with other members of the TLR family (TLR1 or TLR6) [12]. TLR1/TLR2 heterodimers recognize mainly triacylated lipopeptides, whereas TLR2/TLR6 heterodimers recognize diacylated lipopeptides [13], [14]. TLR1/2 heterodimers induce also a different immune response against pathogens as compared to TLR2/6 heterodimers [15], [16]. When TLR1/2 molecules are absent, less induction of early cytokines were observed, whereas TLR2/6 seems able to modulate the balance between a Th1/Th2 immune response.

Limited information is available about recognition of *B. burgdorferi* by TLR1 and TLR6 in murine and human cell systems, and the relative contribution of these receptors as components of the heterodimers with TLR2 for the recognition of *Borrelia* species has not been elucidated for primary cells [7], [17]–[20]. In addition, mutations in TLR1 and TLR6 receptors are associated with differential susceptibility to bacterial and fungal infections [15], [21], and the question arises to what extent these polymorphisms may lead to changes in production of cytokines after exposure to *Borrelia* species, and hence might influence the clinical outcome of Lyme disease. Thus, we investigated the role of TLR1 and TLR6 in the recognition of *Borrelia* species by mouse cells and primary human cells, and assessed whether polymorphisms in either the TLR1 or the TLR6 gene influence the cytokine responses. We observed an important role for TLR1/2 heterodimers for the recognition of *Borrelia* species and for the induction of an early immune response against *Borrelia* spirochetes in humans.

## Results

### TLR1 activation enhanced the induction of IFN-γ by murine splenocytes after exposure to *B. burgdorferi*

**Figure 1:**
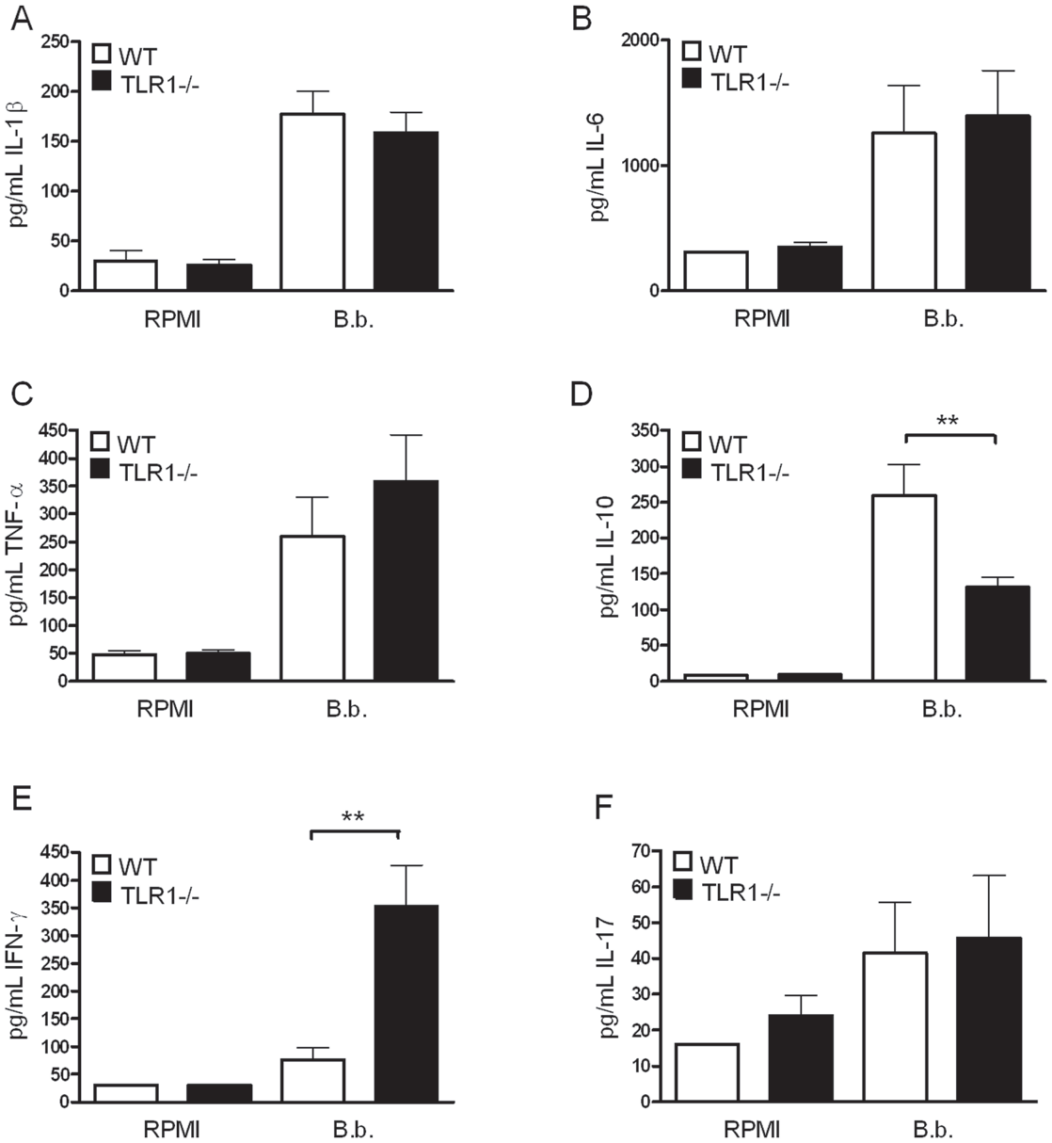
In vitro cytokine production by TLR1−/− cells after stimulation with *Borrelia.* 1×10^5^ peritoneal macrophages from five C57Bl/6 mice were stimulated separately for 24 hours with 1×10^6^ live *B.burgdorferi* per mL. Levels of IL-1β (A), IL-6 (B), and TNF-α (C) were measured in the supernatants and compared to the cytokine production induced by cells deficient in expressing TLR1 (black bars, represent TLR1−/− cytokine responses). Spleen cells (5×10^6^/well) of both wild-type (black bars) and TLR1−/− (white bars) mice were stimulated for 5 days with 1×10^6^ live *Borrelia* per mL and levels of IL-10, IFN-γ, and IL-17 were measured in the supernatant using ELISA (D–F, respectively). Bars represent the mean ± SEM of 5 animals per group. **p<0.01 (for comparisons between wild-type and knock-out mice), Mann-Whitney U-test, experiments were performed in duplicates.

Peritoneal macrophages from TLR1 knockout animals produced the same amounts of pro-inflammatory cytokines IL-1β, IL-6, and TNF-α, as compared to cells isolated from wild-type C57Bl/6 mice (Figure 1A–C). However, a significant decrease in IL-10 production could be observed when TLR1-deficient splenocytes were stimulated with *B. burgdorferi* for 5 days (Figure 1D). Moreover, IFN-γ production induced by *B. burgdorferi* in TLR1-deficient splenocytes was significantly higher than in controls (Figure 1E). Finally, IL-17 production was somewhat higher after stimulation of TLR1 knockout cells, but not found to be statistically significant (Figure 1F).

### *Borrelia*-induced IFN-γ production by murine cells is dependent on TLR6

**Figure 2:**
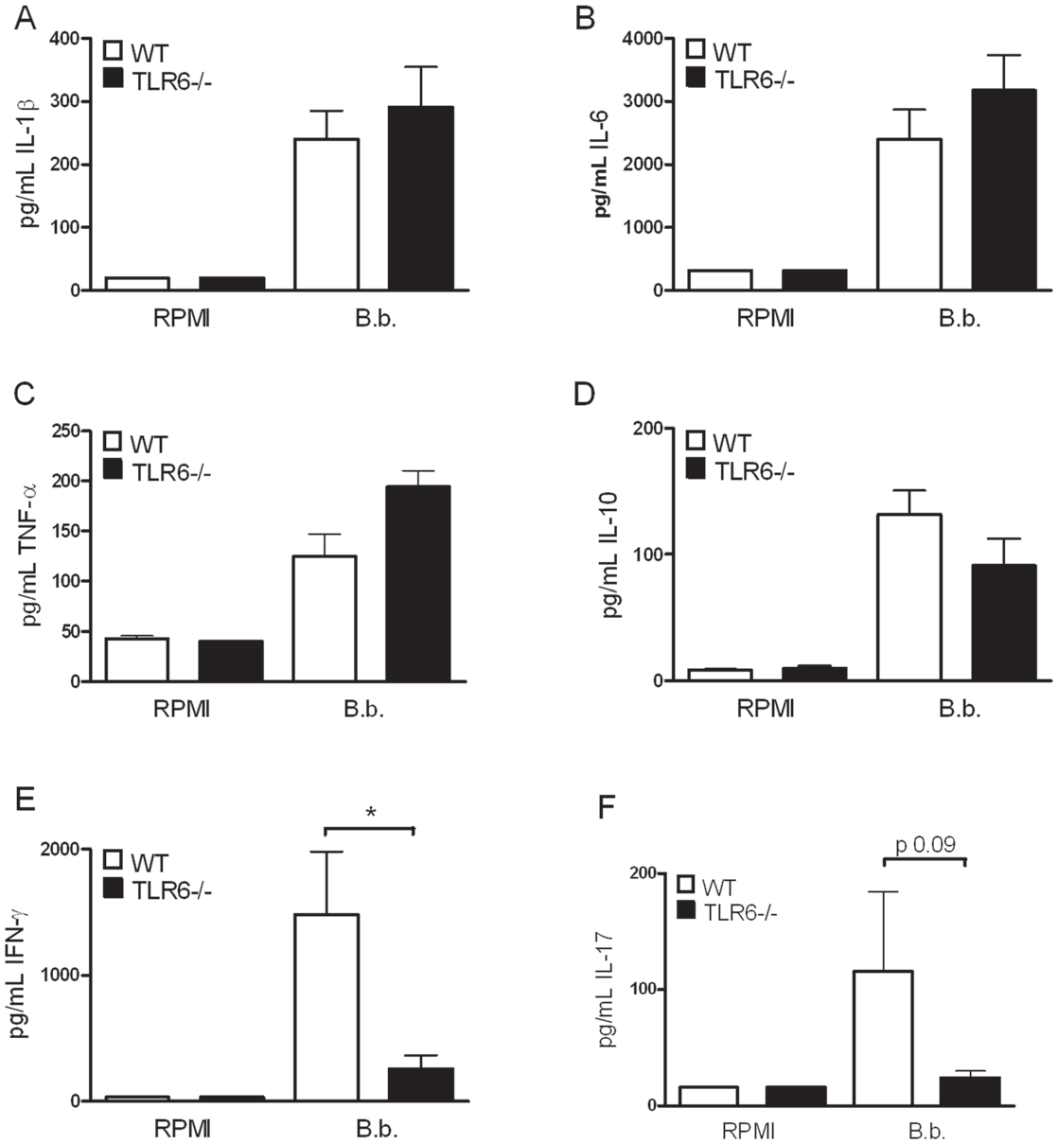
Recognition of *Borrelia* species by immune cells of TLR6−/− mice. Peritoneal macrophages (1×10^5^/well) of C57Bl/6 wild-type or TLR6 gene deficient mice (n = 5 per group) were stimulated for 24 hours with 1×10^6^ live *Borrelia* per mL. Using ELISA or RIA, IL-1β (A), IL-6 (B), or TNF-α (C) levels were measured in pg/mL. 5×10^6^ spleen cells/well were stimulated with 1×10^6^ live *Borrelia* per mL. IL-10, IFN-γ, and IL-17 levels were determined in the supernatant of 5-days spleen cell culture (D–F, respectively). White bars represent cytokine induction after stimulation of wild-type cells, black bars the TLR6 knockout cells. An asterisk indicates that the P-value is <0.05 (for comparisons between wild-type and knock-out mice), 5 animals per group, Mann-Whitney U-test. Bars represent the mean ± SEM, experiments were performed in duplicates.

To assess the role of TLR6, 1×10^6^ live *Borrelia burgdorferi* spirochetes were added to freshly isolated peritoneal macrophages of either wild-type or TLR6 gene-deficient mice. After 24 hours of stimulation, no differences in the production of pro-inflammatory cytokines IL-1β, IL-6, and TNF-α could be detected between cells isolated from wild-type or TLR6 knock-out mice (Figure 2A, 2B, and 2C, respectively). Other cytokines, such as IL-10, IL-17 and IFN-γ are known to be involved in the immune response against *Borrelia* [23], [24], splenocytes of wild-type or TLR6 knock-out mice were incubated for 5 days with *Borrelia.* Thereafter, the production of IL-10, IFN-γ, and IL-17 was measured by ELISA (Figure 2D–F). A significant decrease in IFN-γ production was detected in mice lacking functional TLR6. IL-10 and IL-17 production tended to be lower in these mice, but these differences did not reach statistically significance (Figure 2D and 2F).

### Differential role of human TLR1 and TLR6 for the recognition of *Borrelia*

**Figure 3:**
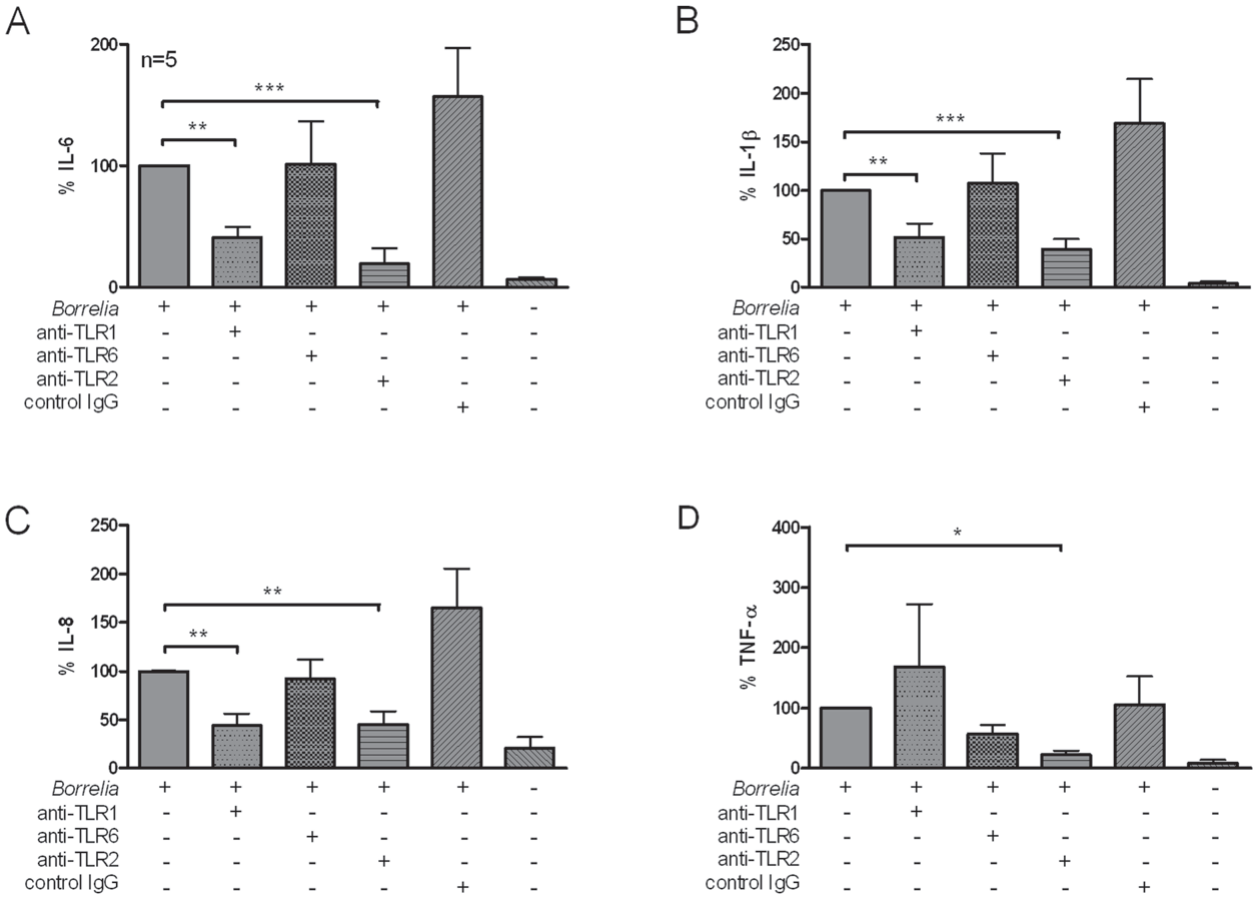
*Borrelia* is recognized by human TLR1 and TLR2. (A) Peripheral blood mononuclear cells (PBMCs, 5×10^5^/well) from 6 healthy volunteers were stimulated for 24 h with 1×10^6^ *B.burgdorferi* per mL (grey bars). IL-6 production in the supernatant was measured using ELISA and showed in % where *Borrelia* induced cytokine production is set as 100% cytokine induction. Bars represent the means ± SEM. **p<0.01; ***p<0.001 (Mann-Whitney). *Borrelia* IL-6 production 100% was 30271±8607 pg/mL. Anti-TLR1, 10 µg/mL specific antibody; anti-TLR6, 10 µg/mL specific antibody; anti-TLR2, 10 µg/mL specific antibody; control IgG, mouse IgG1<$>\scale 80%\raster="rg1"<$> isotype control 10 µg/mL. (B) IL-1β production measured in supernatant after 24 hours culture of PBMCs stimulated with or without 1×10^5^ *B.burgdorferi* per mL or in the presence or absence of 10 µg/mL antibody. *Borrelia* IL-1β production 100% was 576±295 pg/mL. (C) IL-8 production. *Borrelia* IL-8 production 100% was 121±33 ng/mL (D) TNF-α production after 24 hours of stimulation. *Borrelia* TNF-α production 100% was 6528±2716 pg/mL Bars represent the means ± standard error of the means; *p<0.05; **p<0.01; ***p<0.001, Mann-Whitney U-test. The data shown are from three independent experiments each performed in duplicate.

To investigate the relative roles of TLR1 and TLR6 signalling for the recognition of *Borrelia* by human primary cells, neutralizing antibodies were used to inhibit the function of these specific TLRs. When PBMCs from 5 healthy individuals were incubated for 24 hours with blocking anti-TLR1, anti-TLR6, anti-TLR2 antibodies, or control IgG1<$>\scale 80%\raster="rg1"<$> antibody alone, no production of pro-inflammatory cytokines and chemokines could be detected (data not shown). Inhibition of cytokines produced upon Pam3Cys stimulation could only be observed when using the anti-TLR1 antibody and could not be inhibited by TLR6 antibodies (not shown). When human PBMCs were incubated with *B. burgdorferi* in the presence of a specific anti-TLR1 antibody, a significant reduction in IL-1β, IL-6 (but not TNFα), and chemokine IL-8 production was observed (Figure 3A-D). In contrast to TLR1, human TLR6 seems to play a minor role in the induction of pro-inflammatory cytokines after *Borrelia* stimulation of PBMCs. Production of IL-6, IL-1β, or IL-8 was not significantly inhibited after a neutralizing TLR6 antibody was added to the cultures (Figure 3A-D). As demonstrated previously, TLR2 is important for the induction of cytokine responses by *Borrelia* in human PBMCs (Figure 3A-D). No significant differences in cytokine production could be observed when *Borrelia* was co-incubated with IgG antibody alone (Figure 3A–D).

### The role of human TLR1 polymorphisms in cytokine production by *B. burgdorferi*

**Figure 4:**
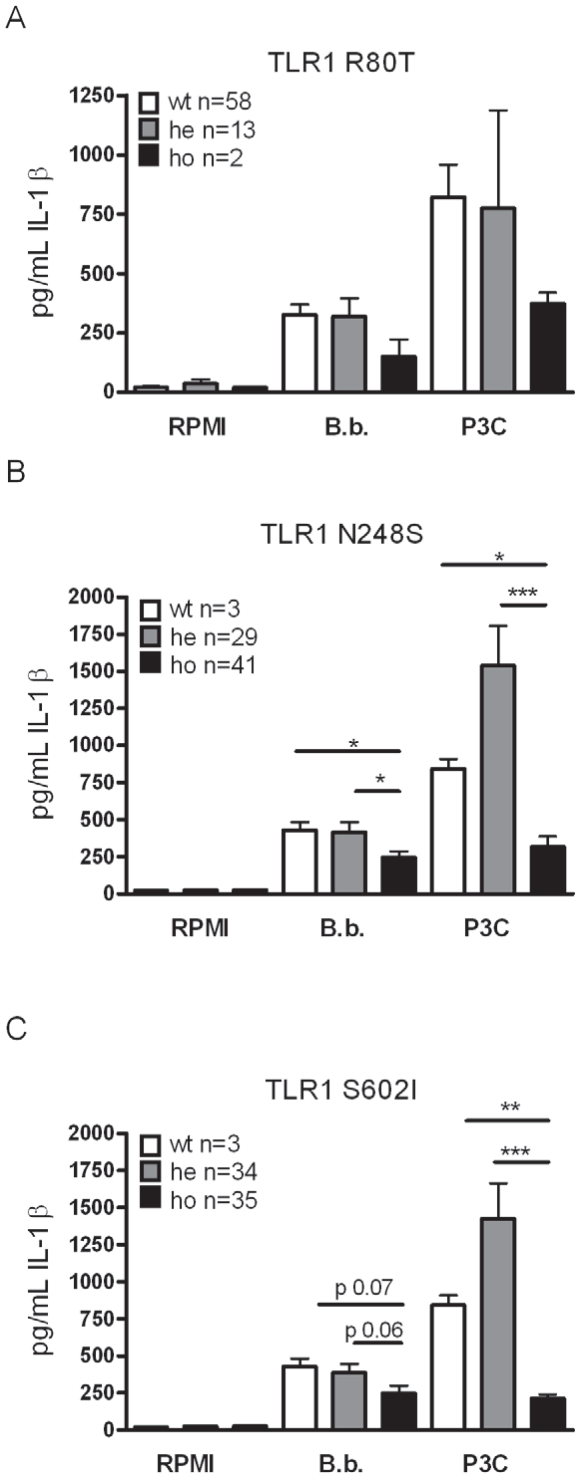
Functional consequences of human TLR1 SNPs in cytokine production. Peripheral blood mononuclear cells (PBMCs) from healthy volunteers were stimulated for 24 h with different stimuli, including *Borrelia burgdorferi* (1×10^6^ spirochetes/mL), and 10 µg/mL Pam3Cys. After stimulation, supernatants were collected, and cytokine levels were measured by enzyme-linked immunosorbent assay. The TLR1 status of these individuals was determined before PBMC stimulation, and they were separated into 3 groups—one group did not displayed the R80T SNP (A), N248S SNP (B), or S602I SNP (C) in TLR1 (white bars; wt; wild-type), one group had a heterozygous mutation (grey bars), and one group had a homozygous mutation (black bars). Data are means ± standard errors. IL-1β, interleukin 1β; RPMI, Roswell Park Memorial Institute 1640 medium; *p<0.05; **p<0.01; ***p<0.001, Mann-Whitney U-test. The data shown are from three independent experiments each performed in duplicate.

PBMCs isolated from individuals carrying different TLR1 genotypes were incubated for 24 hours with *Borrelia,* or the TLR2 specific ligand Pam3Cys. Cytokine production was significantly lower after stimulation of PBMCs with Pam3Cys in cells isolated from individuals homozygous for the R80T, N248S, and S602I polymorphisms in TLR1 (Figure 4). For the TLR1 R80T polymorphism, we observed a trend towards lower production of IL-1β after PBMC stimulation with 1×10^6^ *B*. *burgdorferi* microorganisms per mL (Figure 4A). These results are in line with previous reports showing that the presence of the TLR1 polymorphisms R80T, N248S and S602I led to decreased signaling and cytokine production after Pam3Cys stimulation [23]–[26]. A significantly decreased IL-1β production after *Borrelia* exposure could be detected in persons heterozygous or homozygous for the TLR1 polymorphism N248S or S602I (Figure 4B and 4C). Significant differences between the wild-type, heterozygous, and homozygous carriers of the TLR1 SNPs were found with respect to IL-6, IL-8, TNF-α, and IL-10 production, after incubation of their PBMCs with *Borrelia* or Pam3Cys (Supplementary tables S1, S2, and S3).

### Human TLR6 polymorphisms are less involved in cytokine induction after stimulation with *B. burgdorferi*

**Figure 5:**
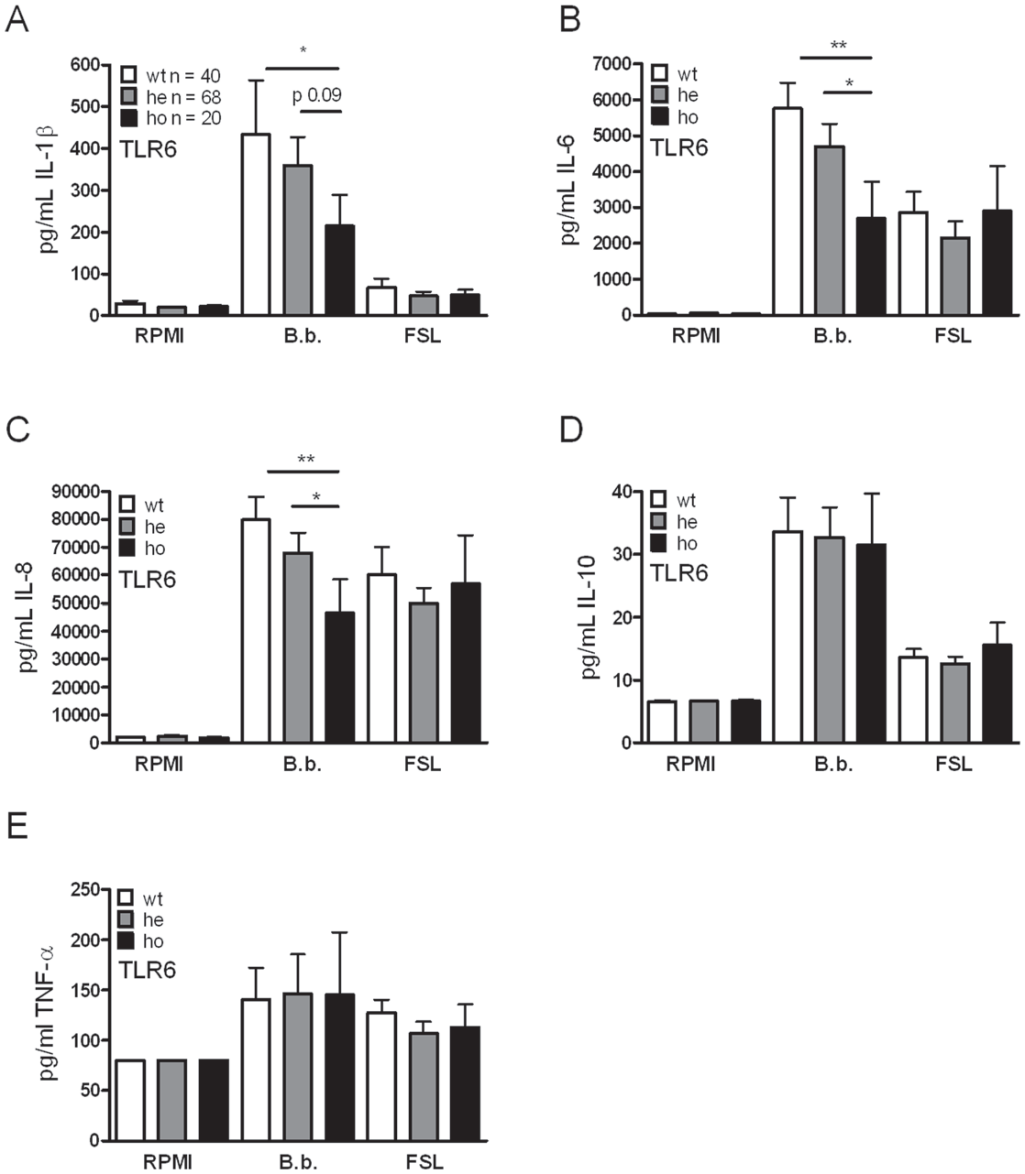
Less important role for human TLR6 in the induction of cytokines after *Borrelia* stimulation. Peripheral blood mononuclear cells (PBMCs) from 128 healthy volunteers carrying the S249N SNP in TLR6 were stimulated with either medium, 1×10^6^ per mL *Borrelia* spirochetes, or 1 µg/mL FSL-1 for 24 h and cytokines were measured using ELISA; (interleukin 1β [IL-1β],(A); IL-6,(B); IL-8,(C); IL-10, (D); and tumor necrosis factor α [TNF-α],(E)). Bars represent individuals carrying no SNP (wild-type, wt, white bars), heterozygous SNP carriers (he, grey bars), or homozygous variation (ho, black bars). Data represent the mean ± SEM, *p<0.05; **p<0.01; Mann-Whitney U-test. The data shown are from three independent experiments each performed in duplicate.

The non-synonymous polymorphism S249P in the TLR6 gene, believed to result in a malfunction in the extracellular domain of the TLR6 molecule, is present with a high frequency in several different populations (>10%) [27]. However, after PBMC stimulation for 24 h with the specific TLR2/6 ligand FSL-1, we did not detect differences between individuals with or without the TLR6 polymorphism in either IL-1β, IL-6, IL-8, IL-10, or TNF-α (Figure 5A-E, respectively). On the other hand, we could observe lower IL-1β, IL-6, and IL-8 production by cells of individuals bearing the S249P SNP after stimulation with *Borrelia burgdorferi*.

### Human TLR1 is involved in *Borrelia*-induced IFN-γ

**Figure 6:**
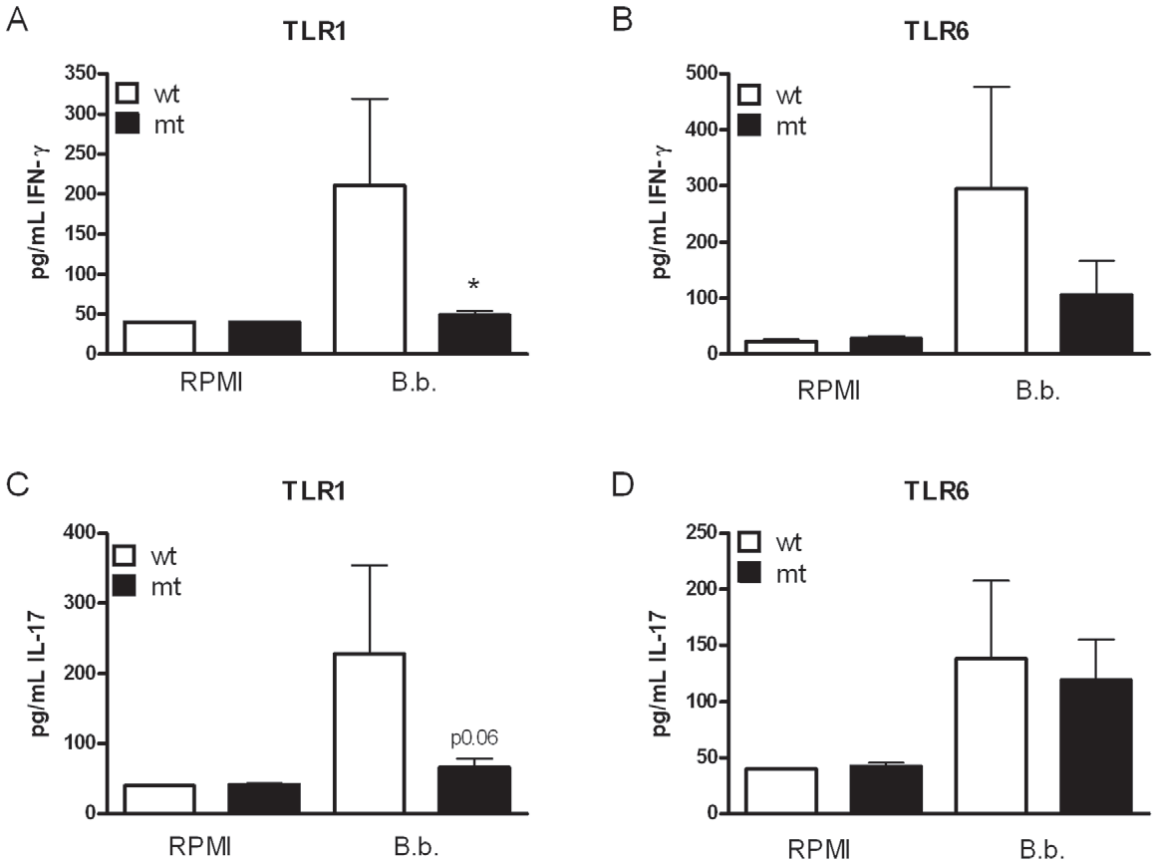
Human TLR1 is involved in *Borrelia-*induced IFN-γ. Peripheral blood mononuclear cells (PMBCs, 5×10^5^/ well) from individuals bearing a SNP in TLR1 (N248S) or TLR6 (S249P) were stimulated with medium, 1×10^6^ *Borrelia* per mL, or Pam3Cys (10 µg/mL), in the presence of 10% human pool serum. After 7 days of incubation, IFN-γ (A, B) and IL-17 (C, D) levels were determined in the supernatant using ELISA. Bars represent individuals carrying no SNP (wild-type, wt, white bars, n≥3 for TLR1 SNPs and n≥5 for TLR6 SNPs), or individuals bearing the SNP in one allele or in both alleles (mutant, mt, black bars, n≥6 for TLR1 SNPs and n≥7 for TLR6 SNPs). Data represent the means ± the standard error of the means, Mann-Whitney U test, *p<0.05. The data shown are from three independent experiments each performed in duplicate.

Since we demonstrated that TLR1 controls the induction of IFN-γ in mice, we assessed whether TLR1 is involved in human IFN-γ and IL-17 responses upon encounter of *Borrelia* species. PBMCs isolated from healthy volunteers bearing either a TLR1 or TLR6 polymorphism were stimulated for 7 days with either medium, live *Borrelia,* or Pam3Cys. Pam3Cys-induced IFN-γ was less produced by cells isolated from individuals bearing TLR1 SNPs, but no differences could be observed for cells with the described TLR6 SNP (data not shown). IFN-γ levels after *Borrelia* stimulation were significantly decreased in individuals lacking a functional TLR1 molecule, whereas TLR6 seems not to play a major role in the induction of this pro-inflammatory cytokine (Fig. 6A-B, respectively). TLR1 might also play a role in the induction of IL-17 after *Borrelia* exposure, a trend towards lower production could be detected, although not found to be significant (Fig.6C). TLR6 seems not involved in *Borrelia-*induced IL-17 production (Fig.6D).

## Discussion

The present study expands the knowledge regarding the role of both TLR1/2 and TLR2/6 heterodimers in the recognition of *Borrelia* spp. We demonstrate that TLR1 in humans is an important component for the recognition and induction of an immune response against *Borrelia*. This was demonstrated by experiments using either TLR1- or TLR6 deficient murine cells, as well as studies performed in cells isolated from individuals bearing genetic polymorphisms in TLR1 or TLR6. Of high interest, a different pattern was found in murine cells; genetic disruption of TLR1 resulted in an upregulated IFN-γ response, whereas TLR6 deficient cells were unable to induce a sufficient IFN-γ response after *Borrelia* exposure. This difference in IFN-γ induction in mice between TLR1 or TLR6 could not be observed in humans. Neither TLR1 and TLR6 seem to be involved in the induction of this cytokine upon stimulation with *Borrelia* spirochetes.

TLR1/2 or TLR2/6 heterodimers do not only differ in structure, but also recognize different ligands. Whereas TLR1/2 heterodimers mainly recognize tri-acetylated structures such as Pam3Cys, di-acetylated proteins are mainly recognized by TLR2/6 heterodimers [13], [14]. It is known that *Borrelia* spp. contains tri-acetylated proteins in the cell membrane and this fits with our observation that the TLR1 containing heterodimers are more important in recognition and signaling of *Borrelia*.

The genome of *Borrelia burgdorferi* encodes at least 105 membrane proteins, including the group of immunologically important outer surface proteins (OSPs) [28]. It has been suggested that *Borrelia* outer surface protein A (OspA) plays an important role in the pathogenesis of Lyme disease caused by *Borrelia* species. Macrophages isolated from TLR1 deficient mice display an impaired ability to induce IL-6 after stimulation with OspA lipoprotein [7]. In contrast to these studies, we were unable to detect any differences in IL-6 production between wild-type and TLR1 knockout mice, when cells were stimulated with intact *Borrelia* spirochetes. This effect might be due to the presence of multiple ligands that are absent in studies that use solely purified OspA protein in supraphysiological concentrations. Using intact bacteria, the immune response is likely stimulated through several distinct PRR pathways, involving both TLRs and NODs [6].

Disruption of either the TLR1 or TLR6 gene in mice did not lead to changes in the pro-inflammatory cytokine production, with the exception of the T-cell-derived IFN-γ and IL-17. This implies that in the mouse the intact TLR1/TLR2 heterodimers dampen the interferon gamma response. This effect may be mediated through IL-10, since this cytokine was downregulated in mice lacking functional TLR1 molecules. It may be hypothesized that murine TLR1 and TLR6 exert counter-regulatory roles for the induction of Th1/Th17 cytokines by *Borrelia* bacteria.

Mutations in TLR1 can lead to a decreased surface protein expression of TLR1 on the cell surface and diminished activation of NF-<$>\scale 80%\raster="rg1"<$>B [25], [26]. It has been previously suggested that defects in TLR1 signaling might play a role in the early induction of the immune response in mice against *Borrelia* spp [7]. As genetic variants have been reported to modify the function of TLRs and influence susceptibility to infections in humans, we assessed the role of three SNPs in TLR1 and one SNP in TLR6 for their role in the induction of cytokines after recognition of *Borrelia* [21], [26]. SNPs in TLR1 impair intracellular trafficking of the TLR1, which eventually leads to a decreased NF-<$>\scale 80%\raster="rg1"<$>B signaling. It was described before that intracellular interactions between TLR1 and TLR2 are necessary for optimal immune signaling in human cells [29]. A different pattern of TLR involvement could be observed in peripheral blood mononuclear cells isolated from humans in comparison to the cytokines induced by mouse cells. Using cells of individuals with functional genetic polymorphisms in the TLR1 gene, we found a marked inhibition of the pro-inflammatory cytokine production when these cells were exposed to *Borrelia.* This was found in individuals carrying the polymorphism in both alleles (homozygous) for any of the three SNPs in TLR1 studied. In agreement with our data, Johnson et al exposed human monocytes with the S602I SNP in the TLR1 gene to TLR1/TLR2 agonists and also observed an blunted pro-inflammatory response [26].

In a recent report, the S602I SNP in TLR1 was linked to the N248S SNP in this gene [25]; also in our study these two SNPs displayed an 80% linkage. However, no linkage was observed with the third SNP in TLR1 (R80T). SNP N248S and R80T have previously been associated with invasive aspergillosis [21], and our finding of modulation of cytokines when cells with these genetic variations are stimulated with *B. burgdorferi* warrants studies of these polymorphisms in patients with Lyme disease.

The presence of the TLR6 S249N SNP did also influence IL-1β, IL-6, and IL-8 production induced by *B. burgdorferi*. TNF-α and IL-10 levels induced by *Borrelia* were not changed in the presence of the SNP. However, it did not influence cytokine responses induced by TLR6/2 ligand FSL-1. Until now, the precise function of this SNP is still unknown, although it has been negatively correlated with ulcerative colitis and the development of clinical signs of malaria, and probably offers protection against the development of asthma [27], [30], [31].

These genetic findings are in agreement with those we obtained in cultures in which human PBMCs were exposed to antibodies against TLR1 or TLR6. In these experiments we found that TLR6 had only a marginal effect on cytokine production (some effect on TNF-α production), whereas TLR1 clearly mediates *Borrelia-*induced cytokine production. TLR1 is also responsible for T-cell derived cytokines after recognition of *Borrelia.* We observed decreased IFN-γ and IL-17 production when TLR1 molecules were dysfunctional through the presence of polymorphisms.

In conclusion, the present study demonstrates an important role for TLR1/TLR2 heterodimers for the recognition of *Borrelia* in humans. Furthermore, the presence of genetic variants of TLR1 gene leads to impaired cytokine responses upon challenge of PBMCs with *Borrelia.* On the one hand, since initiation of host defense responses against *Borrelia* is dependent on multiple pattern recognition receptors, more research is needed to elucidate the precise role of TLR1/2 in the pathogenesis of Lyme disease. On the other hand, these results give novel information regarding the mechanisms of *Borrelia* recognition and the role of TLR1 in this process, and warrants future studies in the role of this receptor for the susceptibility to Lyme disease.

## Materials and Methods

### Borrelia burgdorferi cultures

*B.burgdorferi,* ATCC strain 35210, was cultured at 33°C in Barbour-Stoenner-Kelley (BSK)-H medium (Sigma-Aldrich) supplemented with 6% rabbit serum. Spirochetes were grown to late-logarithmic phase and examined for motility by dark-field microscopy. Organisms were quantitated by fluorescence microscopy after mixing 10 µL aliquots of culture material with 10 µL of an acridine orange solution and counted using a Petroff-Hauser counting chamber. Bacteria were harvested by centrifugation of the culture at 7000 x g for 15 min., washed twice with sterile PBS (pH 7.4), and diluted in the specified medium to required concentrations of 1×10^6^ spirochetes per mL. Heat-killed *B. burgdorferi* were prepared by heating cultured spirochetes at 52°C for 30 min. before dilution.

### Animals

TLR1−/− and TLR6−/− mice were kindly provided by Dr. Shizuo Akira and are fully backcrossed to C57BL/6 background (Osaka University, Japan). C57BL/6 mice were obtained from Charles River Wiga (Sulzfeld, Germany). Female wild-type and knock-out mice between 8 and 14 weeks of age were used. The mice were fed sterilized laboratory chow (Hope Farms, Woerden, The Netherlands) and water *ad libitum*. The experiments were approved by the Ethics Committee on Animal Experiments of the Radboud University Nijmegen Medical Centre.

### *In-vitro* cytokine production

Peritoneal macrophages were isolated by injecting 5 mL of ice-cold sterile PBS (pH 7.4) in the peritoneal cavity. After centrifugation and washing, cells were resuspended in Roswell Park Memorial Institute (RPMI) 1640 containing 1 mM pyruvate, 2 mM L-glutamine and 50 mg/L gentamicin (culture medium). Cells were counted using a Z1 Coulter Particle Counter (Beckman Coulter, Woerden, The Netherlands) and adjusted to 1×10^6^ cells/mL. Cells were cultured in 96-well round-bottom microtiter plates (Costar, Corning, The Netherlands) at 1×10^5^ cells/well, in a final volume of 200 µL. After 24 hours of incubation of cells with different stimuli at 37°C in air and 5% CO_2_, the plates were centrifuged at 1400 x g for 8 min, and the supernatant was collected and stored at −20°C until cytokine assays were performed.

Spleen cells were isolated by gently squeezing spleens in a sterile 200 µm filter chamber. After washing with sterile PBS and centrifugation at 4°C (1200 rpm 5 min), cells were resuspended in 4 ml RPMI 1640 in presence of 20% FCS. Cells were counted and concentrations were adjusted to 1×10^7^ cells/ml. Cells were cultured in 24-wells plates (Greiner, Alphen a/d Rijn, The Netherlands) at 5×10^6^ cells/well, in a final volume of 1000 µl. After 5 days of incubation, supernatant was collected and stored at −80°C until cytokine assays were performed.

### Study populations

Individuals in this study were foresters from the ‘Geldersch Landschap’ and ‘Kroondomein het Loo’ in the Netherlands. In this cohort, Lyme disease occurs as an occupational disease and hence they were invited to participate. The foresters were between 23–73 years old, and consisted of 77% males and 23% females. Samples of venous blood of them were drawn after informed consent was obtained. Experiments were conducted according to the principles expressed in the Declaration of Helsinki.

### Isolation of genomic DNA and single nucleotide polymorphism analysis

DNA was isolated using the Gentra Pure Gene Blood kit (Qiagen), in accordance with the manufacturer's protocol for whole blood. DNA was dissolved in a final volume of 100 µL buffer. Polymerase chain reaction (PCR) amplification of TLR1 and TLR6 gene fragments bearing the polymorphisms R80T (rs5743611), N248S (rs4833095), and S249P (rs5743810) were performed using a pre-designed TaqMan ® SNP genotyping assay (Applied Biosystems) in 25 µL reactions containing 2 µL of genomic DNA as well as primers, two specific probes (with either VIC or FAM label) and Universal PCR 2x Master mix (Applied Biosystems). Cycling conditions were 2 min at 50°C and 10 min at 95°C followed by 40 cycli of 95°C for 15 sec and 1 min at 60°C. Fluorescence intensities were corrected using a post-read / pre-read method for 1 min at 60°C before and after the amplification. The software automatically plotted genotypes based on a two-parameter plot with an overall success rate of more than 95%. Intermediate samples were excluded from the analysis. Conventional PCR amplification of the TLR1 gene fragment containing polymorphism S602I (rs5743618) was performed on the Icycler (BioRad) in 50 µL reactions containing 2 µL of genomic DNA, 1,5 mM MgCl_2_, Taq DNA polymerase conc. 5 U/µL (Invitrogen), PCR buffer, 20 mM dNTP (Pharmacia), 10 µM Forward primer 5′-CTA CCC GGA AAG TTA TAG AGG AAC C, and 10 µM Reversed primer 5′-TTT GGC AAT AAT TCA TTC TTC ACC. PCR consisted of one initial denaturation phase of 95°C for 10 minutes followed by 40 cycles; each cycle consisted out of one annealing step of 95°C for 30 seconds, one polymerization step of 60°C for 30 seconds, and one elongation step of 72°C for 30 seconds. Subsequently, another cycle of 72°C for 7 minutes was performed before termination. PCR products were sequenced with either 4 µM of primers according to the Sanger method supported by Big Dye Terminator version 3 of Applied Biosystems. After sequencing, samples were analyzed using the 3730 Sequence analyzer and Chromas 2.33 software (Technelysium).

### Isolation of human peripheral blood mononuclear cells and *in-vitro* cytokine production

Venous blood was drawn from the cubital vein of foresters into 10 mL ethylenediaminetetraacetic acid (EDTA) tubes (Monoject). Peripheral blood mononuclear cells (PBMCs) were isolated according to standard protocols, with minor modifications. The PBMC fraction obtained by density centrifugation of blood diluted 1∶1 in phosphate-buffered saline (PBS)-buffer over Ficoll-Paque (Pharmacia Biotech). Cells were washed three times in PBS and resuspended in RPMI 1640 (Dutch modified) supplemented with 50 mg/L gentamicin, 2 mM L-glutamin, and 1 mM pyruvate. Cells were counted in a Coulter Counter Z® (Beckman Coulter), and adjusted to 5×10^6^ cells/mL. Mononuclear cells (5×10^5^) in a 100 mL volume were added to round-bottom 96-wells plates (Costar, Corning, The Netherlands) and incubated with either 100 µL of medium (negative control) or *B. burgdorferi (*1×10^6^ spirochetes per mL). In some experiments, PBMCs were pre-incubated with neutralizing antibodies for 30 minutes (functional grade anti-human Toll-like receptor 1 (10 µg/mL, eBioscience), anti-TLR6 (10 µg/mL, BioLegend), anti-TLR2 (10 µg/mL, eBioscience) or control antibody (mouse IgG1<$>\scale 80%\raster="rg1"<$>, 10 µg/mL, eBioscience)). After pre-incubation, *B. burgdorferi* or specific TLR ligands were added, such as Pam3Cys or FSL-1 (10 µg/mL or 1 µg/mL, respectively). After 24 hours or 7 days (in the presence of 10% human pool serum) supernatants were collected and stored at −20°C until being assayed.

### Cytokine measurements

Concentrations of mouse IL-1β were determined by specific radioimmunoassay (RIA; detection limit is 20 pg/mL) [22]. Mouse IL-6, IL-17, IFN-γ, and IL-10 concentrations were measured by a commercial ELISA kit (Biosource, Camarillo, CA; detection limits 16 pg/mL), according to the instructions of the manufacturer. Concentrations of human IL-1β, IL-6, IL-17, or IFN-γ were determined in duplicates using either specific or commercial ELISA kits (PeliKine Compact, Sanquin, Amsterdam, or R&D Systems, Minneapolis), in accordance with the manufacturers' instructions. Detection limits were 40 pg/mL, except for IFN-γ ELISA (12 pg/mL).

### Ethics statement

All experiments in this study were carried out in strict accordance with the recommendations in the Guide for the Care and Use of Laboratory Animals of the National Institutes of Health, the Dutch law on Animal experiments, and FELASA regulations. The protocol was approved by the Ethics Committee on Animal Experiments of the Radboud University Nijmegen Medical Centre. All efforts were made to minimize suffering of the animals.

All human experiments were conducted according to the principles expressed in the Declaration of Helsinki. Before taking blood, informed written consent of each human subject was provided. The study was approved by the review board of the department of Medicine of the Radboud University Nijmegen Medical Centre.

### Statistical Analysis

The data are expressed as mean ± SEM unless mentioned otherwise. Differences between experimental groups were tested using the two-sided Mann-Whitney *U* test performed on GraphPad Prism 4.0 software (GraphPad). *P* values of ≤0.05 were considered significant.

## Supporting Information

Table S1 Cytokine production in pictograms per milliliter after stimulation of PBMCs isolated from healthy volunteers carrying R80T SNP in TLR1 molecules. All values are depicted as means plusminus the standard error of the means.
(DOC)

Table S2 Cytokine production in pictograms per milliliter after stimulation of PBMCs isolated from healthy volunteers carrying N248S SNP in TLR1 molecules. All values are depicted as means plusminus the standard error of the means.
(DOC)

Table S3 Cytokine production in pictograms per milliliter after stimulation of PBMCs isolated from healthy volunteers carrying S602I SNP in TLR1 molecules. All values are depicted as means plusminus the standard error of the means.
(DOC)
