# Supplementary material for: TLR1/TLR2 Heterodimers Play an Important Role in the Recognition of Borrelia Spirochetes
Source: PLoS One. 2011 Oct 5;6(10):e25998. doi: 10.1371/journal.pone.0025998 (PMC3187844; doi:10.1371/journal.pone.0025998)
Supplement: Table S1 — Cytokine production in pictograms per milliliter after stimulation of PBMCs isolated from healthy volunteers carrying R80T SNP in TLR1 molecules. All values are depicted as means plusminus the standard error of the means. (DOC) [file pone.0025998.s001.doc]

**Table S1.** Cytokine production by PBMCs with R80T SNP in TLR1.

|  |  |  | **Averages in pg/mL ± SEM** | | | | |
| --- | --- | --- | --- | --- | --- | --- | --- |
| **SNP** | **Stimulus** | **Group** | **IL-1β** | **IL-6** | **IL-8 (ng/mL)** | **TNF-α** | **IL-10** |
| **R80T** | **RPMI** | Wt | 23 ± 2 | 46 ± 24 | 1.8 ± 0.4 | 80 ± 0 | 7 ± 0 |
|  |  | He | 37 ± 17 | 17 ± 1 | 1.2 ± 0.2 | 80 ± 0 | 7 ± 0 |
|  |  | Ho | 20 ± 0 | 15 ± 0 | 0.6 ± 0.07 | 80 ± 0 | 7 ± 0 |
|  | **B.burgdorferi** | Wt | 326 ± 44 | 5327 ± 755 | 72.2 ± 8.0 | 154 ± 31 | 32 ± 4 |
|  |  | He | 319 ± 76 | 4455 ± 1470 | 59.8 ± 14.8 | 113 ± 20 | 22 ± 5 |
|  |  | Ho | 151 ± 124 | 770 ± 530^a^ | 13.5 ± 4.5^a^ | 80 ± 0 | 7 ± 0^a^ |
|  | **Pam3Cys** | Wt | 821 ± 133 | 10110 ± 1072 | 15.1 ± 10.7 | 289 ± 40 | 187 ± 25 |
|  |  | He | 777 ± 394 | 6197 ± 1829 | 116.3 ± 23.2 | 209 ± 66 | 156 ± 57 |
|  |  | Ho | 373 ± 83 | 7150 ± 4250 | 160.5 ± 80.5 | 210 ± 60 | 60 ± 6 |

**^a^ Wt versus Ho p<0.05; ^b^ Wt versus Ho p<0.01; ^c^ He versus Ho p<0.05; ^d^ He versus Ho p<0.01; ^e^ He versus Ho p<0.001**
