# Supplementary material for: TLR1/TLR2 Heterodimers Play an Important Role in the Recognition of Borrelia Spirochetes
Source: PLoS One. 2011 Oct 5;6(10):e25998. doi: 10.1371/journal.pone.0025998 (PMC3187844; doi:10.1371/journal.pone.0025998)
Supplement: Table S3 — Cytokine production in pictograms per milliliter after stimulation of PBMCs isolated from healthy volunteers carrying S602I SNP in TLR1 molecules. All values are depicted as means plusminus the standard error of the means. (DOC) [file pone.0025998.s003.doc]

**Table S**3. Cytokine production by PBMCs with S602I SNP in TLR1.

|  |  |  | **Averages in pg/mL ± SEM** | | | | |
| --- | --- | --- | --- | --- | --- | --- | --- |
| **SNP** | **Stimulus** | **Group** | **IL-1β** | **IL-6** | **IL-8 (ng/mL)** | **TNF-α** | **IL-10** |
| **S602I** | **RPMI** | Wt | 20 ± 0 | 16 ± 1 | 2.1 ± 0.7 | 80 ± 0 | 7 ± 0 |
|  |  | He | 23 ± 3 | 22 ± 4 | 2.4 ± 0.6 | 80 ± 0 | 7 ± 0 |
|  |  | Ho | 27 ± 6 | 20 ± 4 | 1.1 ± 0.1 | 80 ± 0 | 7 ± 0 |
|  | **B.burgdorferi** | Wt | 428 ± 55 | 7167 ± 784 | 95.3 ± 6.9 | 80 ± 0 | 70 ± 13 |
|  |  | He | 386 ± 61 | 7170 ± 1092 | 91.7 ± 11.5 | 185 ± 48 | 36 ± 6 |
|  |  | Ho | 250 ± 50 | 2600 ± 615^a,e^ | 41.3 ± 7.3^a,e^ | 111 ± 21 | 20 ± 4^a,c^ |
|  | **Pam3Cys** | Wt | 842 ± 67 | 20167 ± 2146 | 232.7 ± 32.3 | 187 ± 44 | 387 ± 155 |
|  |  | He | 1427 ± 227 | 14170 ± 1269 | 196.6 ± 13.0 | 421 ± 63 | 270 ± 31 |
|  |  | Ho | 212 ± 29^b,e^ | 3607 ± 552^b,e^ | 92.8 ± 8.8^b,e^ | 143 ± 16 | 56 ± 8^b,e^ |

**^a^ Wt versus Ho p<0.05; ^b^ Wt versus Ho p<0.01; ^c^ He versus Ho p<0.05; ^d^ He versus Ho p<0.01; ^e^ He versus Ho p<0.001**
